# Synergistic effects and related bioactive mechanism of *Potentilla fruticosa* L. leaves combined with *Ginkgo biloba* extracts studied with microbial test system (MTS)

**DOI:** 10.1186/s12906-016-1485-2

**Published:** 2016-12-01

**Authors:** Ze-Hua Liu, Dong-Mei Wang, Su-Fang Fan, Deng-Wu Li, Zi-Wen Luo

**Affiliations:** College of Forestry, Northwest A & F University, Yangling, Shaanxi 712100 China

**Keywords:** *Potentilla fruticosa L*. leaves, *Ginkgo biloba* extracts, Synergistic mechanism, Microbial test system (MTS), Real-time PCR

## Abstract

**Background:**

*Potentilla fruticosa*, also called “Jinlaomei” and “Gesanghua”, is widely used as folk herbs in traditional Tibetan medicine in China to treat inflammations, wounds, certain forms of cancer, diarrhoea, diabetes and other ailments. Previous research found *P. fruticosa* leaf extract (C-3) combined with *Ginkgo biloba* extracts (EGb) showed obvious synergistic effects in a variety of oxidation systems. The aim of the present study was to further confirm the synergy of *P. fruticosa* combined with EGb viewed from physiological bioavailability and explore the related bioactive mechanism behind the synergism.

**Methods:**

The microbial test system (MTS) was adopted to evaluate the related bioactive mechanism. The synergistic effects were evaluated by isobolographic analysis. The H_2_O_2_ production rate and antioxidant enzyme (Catalase (CAT), Peroxidase (POD), Superoxide dismutase (SOD), Glutathione peroxidase (GSH-PX)) activities were determined by the colorimetric method. Enzyme gene (CAT, SOD) expression was measured by real time-PCR.

**Results:**

The MTS antioxidant activity results showed the combination of C-3 + EGb exhibited synergistic effects especially at the ratio 5:1. Components of isorhamnetin and caffeic acid in C-3 and EGb displayed strong antioxidant activities on MTS and their combination also showed significant synergy in promoting H_2_O_2_ production. The combinations of C-3 + EGb and isorhamnetin + caffeic acid promoted CAT and SOD enzyme activities and the ratio 1:1 exhibited the strongest synergy while no obvious promotion on POD and GSH-PX enzyme activities was found. Both combinations above promoted gene expression of CAT and SOD enzymes and the ratio 1:1 exhibited the strongest synergy.

**Conclusions:**

Antioxidant activity results in MTS further confirmed the significant synergy of C-3 combined with EGb and isorhamnetin combined with caffeic acid. The synergy of C-3 combined with EGb may be attributed to the combination of isorhamnetin + caffeic acid, which promoted CAT and SOD enzyme gene expression and further promoted the enzyme activities in *E. coli.* This study could further provide rational basis for optimizing the physiological bioavailability of *P. fruticosa* by using natural and safe antioxidants in low doses to produce new medicines and functional products.

## Background

Previous research has revealed that many plant antioxidants can protect against oxidation damage and they may even have potential applications in preventing various diseases [1]. Their human use has a good safety record when consumed in reasonable amounts, however, when used in large amounts the safety effects are unknown [2]. Considering the high values of plant antioxidants and their related products, a detailed quantitative analysis of the phytochemical profiles and biological properties, especially of antioxidant combinations with known synergistic effects seem to be a useful exercise.

*P. fruticosa* belonging to the rosaceae family, widely distributed in cool temperate, high altitude regions of the northern hemisphere [3]. In China, the plant species *P. fruticosa* also called “Jinlaomei” and “Gesanghua”, has long been utilized as folk herbs especially in traditional Tibetan medicine for its superior therapeutic effects [4, 6]. The leaf extracts of *P. fruticosa* were used to prevent cardiac-cerebral vascular disease, the stem extracts of *P. fruticosa* were used to treat inflammation, dyspepsia and edema in traditional Tibetan medicine [7–9]. It was also reported that the curative properties of *P. fruticosa* were used to treat certain types of cancer; bacterial, fungal and viral infections; diabetes and other ailments [10–17]. Furthermore, the extracts of *P. fruticosa* possess relatively high concentrations of phenolic acids and flavonoids and powerful radical scavenging capacity, which was higher than that of butylated hydroxytoluene [18–20]. Meanwhile, *Gingko biloba* extracts have gained acceptance in industry as a promising source of antioxidant compounds and the radical scavenging capacities of *P. fruticosa* extracts were even higher than that of the synthetic antioxidant butylated hydroxytoluene (BHT) used as antioxidant in pharmaceutical preparations and cosmetic formulations [21–23].

In consideration of the rational basis for the use of phytochemicals in *P. fruticosa* and EGb were still poorly explored by chemical methods including DPPH, ABTS, FRAP, and the oxygen radical absorbance capacity (ORAC) assay, a strategy to enhance the physiological bioavailability of *P. fruticosa* combined with EGb seems to be imperative [4, 24–26]. According to the results of previous study, the combination of *P. fruticosa* and EGb showed enhanced activity in achieving a superior synergism outcome directed at the free radical oxidant damage. Among the combinations of the 6 phenolic compounds (catechin, caffeic acid, hyperoside, rutin, ellagic acid and quercetin) in *P. fruticosa* and the 4 phenolic compounds (catechin, quercetin, kaempferide and isorhamnetin) in EGb detected by chromatographic fractionation, isorhamnetin combined with caffeic acid displayed the greatest synergistic effects [6]. However, the observation in antioxidant synergistic effects of *P. fruticosa* combined with EGb is still not enough to guide in-depth pharmaceutical applications of *P. fruticosa*. This experiment carried on the modified and improved assay based on microbial test system (MTS) in our lab [27], focused on the physiological bioavailability to further confirm the synergistic effects of *P. fruticosa* combined with EGb and try to find specific combinations with least concentrations that exhibit synergistic effects. Those compounds may help to produce natural and safe antioxidants products in lower doses instead of synthetic antioxidants.

In this consideration, the objectives for this study were: (1) to further confirm the antioxidant synergistic effects of *P. fruticosa* (C-3) combined with EGb studied with MTS assay viewed from physiological bioavailability; (2) to evaluate the antioxidant enzyme activities and gene expression to further explore the related bioactive mechanism of *P. fruticosa* (C-3) combined with EGb; (3) to provide rational basis to optimize the antioxidant capacity and physiological bioavailability of *P. fruticosa* using natural and safe antioxidants in low doses instead of synthetic antioxidants, which might be useful to produce new medicines and functional products.

## Methods

### Plant materials and preparation on the C-3 extracts of *P. fruticosa*

*P. fruticosa* leaves were collected from Huzhu Northern Mountain, Qinghai during 2014 at an altitude of 2940 m (E 102°21.149′, N 36°55.807′) [5]. The voucher specimen was identified by the professor Dengwu Li and was deposited at Herbarium of the Northwest A&F University, Yangling, China (WUK0780381). The crude extracts of *P. fruticosa* were the acetone extraction phase (C-3) extracted by Wang in our lab [6]. The air-dried, ground leaves of *P. fruticosa* were extracted with 80% acetone for three days at room temperature to obtain the crude acetone extracts. Part of them were further partitioned with ethyl acetate extracts and then subjected to column chromatography (15 cm diameter, 120 cm length) with silica gel as stationary phase. The elution of components present in the extract was then dissolved in different concentration gradient of ether/acetone (4:1, 1:1, 1:5 and 0:1 v/v). The third fraction labeled as C-3 was found to be most active in the assay, so the C-3 fraction was selected for further research. Both of the C-3 and EGb extracts were dissolved in the initial concentration of 5 mg/ml and the individual compounds were dissolved in the concentration of 1 mM.

### Bacteria and chemicals

*E.coli* (ATCC No.25922) (Microbial Culture Collection Center of Guangdong Institute of Microbiology, China); *Ginkgo biloba* extracts, EGb 761, standard at European Pharmacopeia (Shanghai Youxin Biological Science and Technology Co., Ltd. PR China); quercetin, catechin, caffeic acid, rutin, hyperoside, kaempferide, ellagic acid and isorhamnetin (Shanghai Yuanye Industrial Co., Ltd. PR China); sodium dihydrogen phosphate, disodium hydrogen phosphate, sodium chloride, potassium chloride, potassium dihydrogen phosphate, hydrogen peroxide 30% (Guangdong Guanghua Chemical Factory Co., Ltd. PR China), dimethyl sulfoxide (DMSO) (Tianjin Bodi Chemical Factroy Co., Ltd. PR China), horseradish peroxidase; Yeast extract, Tryptone (OXOID Ltd., Basingstoke, Hampshire, England). All reagents and solvents used were of analytical grade. Deionized water (0.055 μS/cm) was used to prepare aqueous solutions.

### Determination of antioxidant synergy effects on MTS

To circumvent the limitations of the assays based on chemical mechanism to determine antioxidant synergistic effects, the MTS assay was adopted to measure the bioavailability behind synergism with some modification [27]. Bacteria were cultivated aerobically overnight in LB medium in shaking bed (37 °C, 120 rpm). Cell growth was monitored by measurement of the optical density at 600 nm. One milliliter of cell suspension added to 9.0 ml of LB medium to final OD_600_ = 0.250 ± 0.004. Subsequently, 100 μL of sample (5 mg/ml), 1 ml of the diluted cell suspension and 8.9 ml LB medium were mixed and incubated at 37 °C (180 rpm, 1.5 h).121 After the addition of 6.0 mM H_2_O_2_ we calculated the μ value at 30 min to assess the antioxidant activity of extracts*.* The specific growth rate for each sample was calculated according to the following equation:

$$ \upmu =\mathrm{In}\left(\frac{N}{N_0}\right)/t $$

Where μ was the specific growth rate, N_0_ and N were the optical density at time zero and t. The protective activity of each sample was calculated as follows: the specific growth rate of the *E. coli*-containing samples and 6.0 mM H_2_O_2_ was divided by the specific growth rate of the samples containing only H_2_O_2_. All measurements were done in triplicate.

### Measurement of H_2_O_2_ production rate

Rate of hydrogen peroxide production in phosphate medium was assayed by Amplex Red-horseradish peroxidase detecting system (AR/HRP) [28]. The solutions of studied compounds was prepared freshly. One milligram of AR was dissolved in 0.78 ml of DMSO, and 0.75 ml of this solution was then diluted into 18 ml of 50 mM potassium phosphate (KPi, pH 7.8) to generate a 200 μM stock solution, which was shielded from light. HRP was dissolved in 50 mM KPi (pH 7.8) to 0.02 mg/ml. In order to measure H_2_O_2_, 1 ml sample was mixed with 20 μL HRP and 200 μL AR. H_2_O_2_ concentration in samples was measured using a Shimadzu RF Mini-150 fluorometer (λ_ex_563 nm and λ_em_587 nm) at 0 and 15 min after incubation had started. Note that a small amount of H_2_O_2_ is generated by the dye/HRP detection system itself, this amount was accounted for by the standard curve.

### Preparation of crude enzyme liquid

Bacteria suspension was put into ultrasonic cell crusher and set the parameter as: 5 s chill time, 2 s cell broke time, 11 min total work time, 15 °C liquid temperature. The instrument power must be guaranteed at 350 Hz. Centrifuge the cell disruption liquid for 8000 rpm in 8 min and filter to get the solution.

### Determination of enzyme activities

#### Catalase (CAT) enzyme

CAT enzyme activity was assayed according to the method of Beers with some modification [29]. Reaction system consists of the following substances: 3 ml PBS buffer, 0.1 ml 300 mM/L H_2_O_2_, 0.5 ml crude enzyme liquid. Add the PBS buffer into the control groups. Immediately detect absorbance values under 240 nm in every 30 s for 2 minutes. Take the 0.1 decrease of OD_240_ in one minute for one unit of enzyme activity. CAT enzyme activity was calculated according to the following equation:

$$ \mathrm{Y}\left(\mathrm{U}\cdot {\mathrm{g}}^{-1}\cdot { \min}^{-1}\right)=\frac{\varDelta {\mathrm{A}}_{240}\times {\mathrm{V}}_{\mathrm{t}}}{0.1\times {\mathrm{V}}_{\mathrm{s}}\times \mathrm{t}\times {\mathrm{F}}_{\mathrm{m}}} $$

Above the formula,

$$ {\Delta \mathrm{A}}_{240}={A}_0-\frac{{\mathrm{A}}_{\mathrm{S}1}+{\mathrm{A}}_{\mathrm{S}2}}{2} $$

A_0_ was the initial absorbance, A_S1_, A_S2_ were the sample absorbance, V_t_ was the enzyme liquid volume, F_m_ was sample mass, t was the total time of detection (2 min).

#### Peroxidase (POD) enzyme

The method to determinate the activity of POD enzyme was performed according to Huang with some modification [30]. All measurements were done in triplicate. Substances followed: 2 ml 0.3% H_2_O_2_, 1 ml 0.2% guaiacol, 0.5 ml crude enzyme liquid and 1 ml PBS buffer were added into the reaction system. The PBS buffer was added into the control groups instead. Put the reaction system into water bath for 10 min then add the metaphosphate termination reaction. The reaction value was monitored under the 470 nm absorbance before and after water bath. POD enzyme activity was calculated according to the following equation:

$$ \mathrm{Y}\left(\mathrm{U}\cdot {\mathrm{g}}^{-1}\cdot { \min}^{-1}\right)=\frac{{\Delta \mathrm{A}}_{470}\times {\mathrm{V}}_{\mathrm{t}}}{0.01\times \mathrm{m}\times {\mathrm{V}}_{\mathrm{s}}\times \mathrm{t}} $$

Above the formula, ∆A_470_ was the change of the absorbance during reaction, m was the sample mass, V_t_ was the total volume of enzyme liquid, V_s_ was the consuming enzyme liquid volume of reaction, t was the response time.

#### Superoxide dismutase (SOD) enzyme

The method to determinate the activity of SOD enzyme was performed according to Weng with some modification [31]. Included in the reaction system, there were 0.5 ml Met (130 mmol/L), 0.5 ml NBT (750 μmol/L), 0.5 ml EDTA•Na_2_ (100 μmol/L), 0.5 ml riboflavin (20 μmol/L), 1 ml PBS buffer and 0.5 ml crude enzyme liquid. The tubes were exposed evenly under 4000 xl fluorescent for 20 min, and then monitored under 560 nm absorbance. The 50% nitroblue tetrazolium reduction inhibition under light was taken as one unit of enzyme activity. SOD enzyme activity was calculated according to the following equation:

$$ \mathrm{Y}\left(\mathrm{U}\cdot {\mathrm{g}}^{-1}\cdot { \min}^{-1}\right)=\frac{\left({\mathrm{A}}_t-{\mathrm{A}}_{\mathrm{s}}\right)\times {\mathrm{V}}_{\mathrm{s}}\times \mathrm{V}}{0.5\times {\mathrm{A}}_{\mathrm{ck}}\times {\mathrm{V}}_{\mathrm{t}}\times \mathrm{t}\times {\mathrm{F}}_{\mathrm{m}}} $$

Above the formula, A_t_ was the absorbance of the control group, A_s_ was the sample absorbance, V_t_ was the volume of samples, V_s_ was the total volume of enzyme liquid, t was the response time.

#### Glutathione peroxidase (GSH-PX)

GSH-PX enzyme activity of the tested compounds was assayed by the method described by Xing with some modification [32]. Reaction system consists of the following substances: 0.5 ml GSH-PX standard substances, 1.5 ml double distilled water, 2 ml PBS buffer and 0.1 ml TDNB. All measurements were done in three parallel and the PBS buffer was added into control groups. The samples were reacted for 5 min at room temperature, then the optical absorbance was monitored under 412 nm. GSH-PX enzyme activity was calculated according to the following equation:

$$ \mathrm{Y}\left(\upmu \cdot {\mathrm{g}}^{-1}\mathrm{F}\mathrm{W}\right)=\frac{{\mathrm{C}\times \mathrm{V}}_{\mathrm{t}}}{{\mathrm{V}}_{\mathrm{s}}\times {\mathrm{F}}_{\mathrm{m}}} $$

Above the formula, C was the GSH-PX enzyme concentration in samples calculated from the standard curves, V_t_ was the total volume of enzyme liquid, V_s_ was the volume of samples, F_m_ was the sample mass.

### CAT and SOD gene expression by real-time PCR

Gene sequences of CAT and SOD enzyme from *E. coli* were obtained from the GenBank database and gyrB was used as a reference gene. The primers were designed by Primer premier 5 and Oligo 6.0. The primer sequence of CAT enzyme is: forward primer: 5′-TGGAGTGAATACCACGACGAT-3′, reverse primer: 5′-CATGGAAGC CATCACAAACG-3′, product size is 286 bp; The primer sequence of SOD_a_ is: forward primer: 5′-CCCTGCCATCCCTGCCGTAT-3′, reverse primer: 5′-GTGACC GCCAGCGTTGTTGC-3′, product size is 227 bp; The primer sequence of SOD_b_ is: forward primer: 5′-TCACTACGGCAAGCACCA-3′, reverse primer: 5′- CAGG CAGTTCCAGTAGAAAGTA-3′, product size is 166 bp; The primer sequence of SOD_c_ is: forward primer: 5′-CCAGCGGTTTAGGTTGAT-3′, reverse primer: 5′-TGA AGGGCCAGAAGGTG-3′, product size is 179 bp; The primer sequence of gyrB is: forward primer: 5′-CGGAATGTTGTTGGTAAAGC-3′, reverse primer: 5′-CGTGACGGCAAAGAAGAC-3′, product size is 198 bp.

Total RNA samples from *E. coli* strains were extracted by total RNA isolation kit and treated with DNaseI (Beijing Kang Wei, Biotechnology Industrial Co., Ltd. PR China) to remove genomic DNA contamination. Reverse transcription was performed in a total volume of 20 μL with HiFiScript cDNA first chain synthesis kit (Beijing Kang Wei, Biotechnology Industrial Co., Ltd. PR China). The reaction system contains 4 μL dNTP Mix (2.5 mM each), 2 μL Primer Mix, 2 μL RNA Template, 4 μL 5 × RT Buffer, 2 μL DTT (0.1 M), 1 μL HiFiScript (200 U/μL) and 5 μL RNase-Free Water. The condition was followed by 42 °C for 40 min, 85 °C for 5 min, as recommended by the manufacturer.

Real-time PCR reactions were performed with Ultra SYBR Mixture, using IQ5 fluorescence quantitative analysis software. The thermal cycling conditions comprised an initial step at 94 °C for 4 min, followed by 40 cycles at 94 °C for 30 s, 55 °C for 30 s, and 72 °C for 30 s and then 61 cycles of temperature programming at 65 °C. The change in fluorescence of Ultra SYBR Mixture in every cycle was monitored by the system software, and the threshold cycle (CT) was measured. Gene expression level of the cells exposed to FLC relative to that of the control cells was calculated using the formula 2-∆∆CT, where ∆CT was the CT value of genes of interest minus that of the internal control, and ∆∆CT was the mean ∆CT value of the cells exposed to FLC and/or BBR minus that of the control cells. Reactions were performed in triplicate with independent RNA isolations.

### Isobolographic analysis

The isobolographic analysis was performed according to the method described by Wang in our lab with some modification [6]. To show the interactions (synergistic, additive or antagonistic), all extracts were prepared at 5 mg/ml and individual compounds at 10 mmol/L in various combinations (ratios of 5:1, 3:1, 1:1, 1:3 and 1:5, v/v). If the observed dose of combination was on the additivity isobole or close to it, this indicates no interaction; measurements below and above the additivity isobole indicates antagonism and synergism, respectively [33–37]. In addition, the interaction index, denoted γ, was introduced to further measure the effects of different combinations:

$$ \left(\mathrm{a}/\mathrm{A}\right)+\left(\mathrm{b}/\mathrm{B}\right)=\upgamma $$

Where A and B are the doses of drug A (alone) and B (alone), respectively, that give the specified effect and (a, b) is the combination dose that produces this effect level. If γ = 1, the interaction is additive, if γ < 1, it is antagonistic, and if γ > 1, it is synergistic.

### Statistical analysis

Expected values (E) were calculated as the average of individual observed amounts for each one of two combined extracts or compounds, and observed values(O) came through the observed amounts for combined extracts or compounds [38, 39]. All results were expressed as the mean ± standard deviation (SD). The significant difference was calculated by SPSS 20.0 one-way ANOVA followed by Duncan’s test; values < 0.05 were considered to be significant. All diagrams were draw by Sigmaplot 12.0 and Photoshop 8.0.

## Results and discussion

### MTS antioxidant activity of C-3, EGb and their combinations

Previous research of Wang [6] found that phase C-3 was the most effective fraction isolated from *P. fruticosa* extracts which could be selected as the main section to explore the synergy mechanism. Therefore, C-3 was combined with EGb to produce natural powerful combined antioxidants of *P. fruticosa.* The protective effects of C-3, EGb and their combinations on MTS was first assessed and quercetin was used as the positive control.

**Fig. 1:**
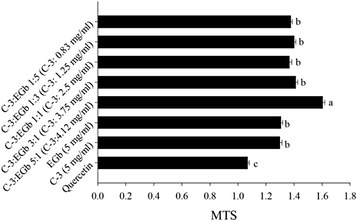
Synergistic effect of C-3, EGb and C-3 combined with EGb on MTS antioxidant activity

The results showed that C-3, EGb and their combinations could better increase cell growth rate than quercetin under oxidative stress. All combinations of C-3 + EGb exhibited synergistic effect and the ratio 5:1 showed strongest synergy with μ_30_ value of 1.603 ± 0.017, followed by 3:1 (1.411 ± 0.016), 1:3 (1.400 ± 0.012), 1:5 (1.376 ± 0.010), and 1:1 (1.366 ± 0.018) (Fig. 1).

In summary, C-3 combined with EGb displayed synergistic effects on MTS and the ratio 5:1 (C-3: EGb) displayed the strongest synergistic, which was in accordance with the results showed in Wang’s research. The results showed that the combination of C-3 + EGb displayed significant antioxidant activity in *E.coli* viewed from bioavailability as well as in antioxidant assays based on chemical methods. This further suggested that EGb could be selected to promote the antioxidant capacity of *P. fruticosa* in order to produce natural powerful combined antioxidants.

### MTS antioxidant activity of individual compounds in C-3 and EGb

To identify above-mentioned phenomena, further study in MTS antioxidant activity of individual compounds in C-3 (catechin, caffeic acid, hyperoside, rutin, ellagic acid and quercetin) and EGb (catechin, quercetin, kaempferide and isorhamnetin) were carried out to explore the mechanism behind the synergism. It is previously reported that these compounds showed relatively high antioxidant capacities respectively [40].

**Fig. 2:**
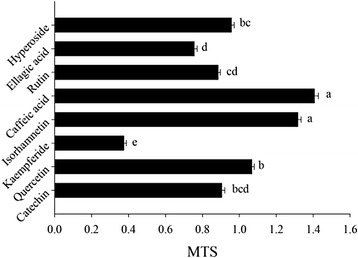
Antioxidant activity of catechin, quercetin, kaempferide, isorhamnetin, caffeic acid, rutin, ellagic acid and hyperoside on MTS

The protective effect of individual compounds, as determined on MTS, was presented in Fig. 2. Among the main phenolics in C-3 and EGb, caffeic acid and isorhamnetin showed a notable protective effect with μ_30_ values of 1.407 ± 0.021 and 1.317 ± 0.017, which were higher than those of the other metabolites.

Since the individual compounds of caffeic acid and isorhamnetin displayed significant synergistic effects on MTS, the protective effects in MTS was likely a result of the existing phytochemicals therein C-3 and EGb. Therefore, isorhamnetin and caffeic acid may played a great role in the synergy observed in the combination of C-3 + EGb, and the results further suggests that the related bioactive mechanism of *Potentilla fruticosa L.* leaves combined with *Ginkgo biloba* extracts may linked to the reaction of individual compounds therein the C-3 and EGb.

### Synergistic effects of C-3 with 4 compounds in EGb on MTS antioxidant activity

The results above-mentioned suggested that the synergistic protective effect was likely due to the high activity as revealed in isorhamnetin and caffeic acid. What’s more, currant literature observed that synergistic actions displayed when crude extracts combined with natural antioxidants [36, 38]. Thus the C-3 was added to the other 4 compounds in EGb on MTS and the protective effects of the combinations was analyzed.

**Fig. 3:**
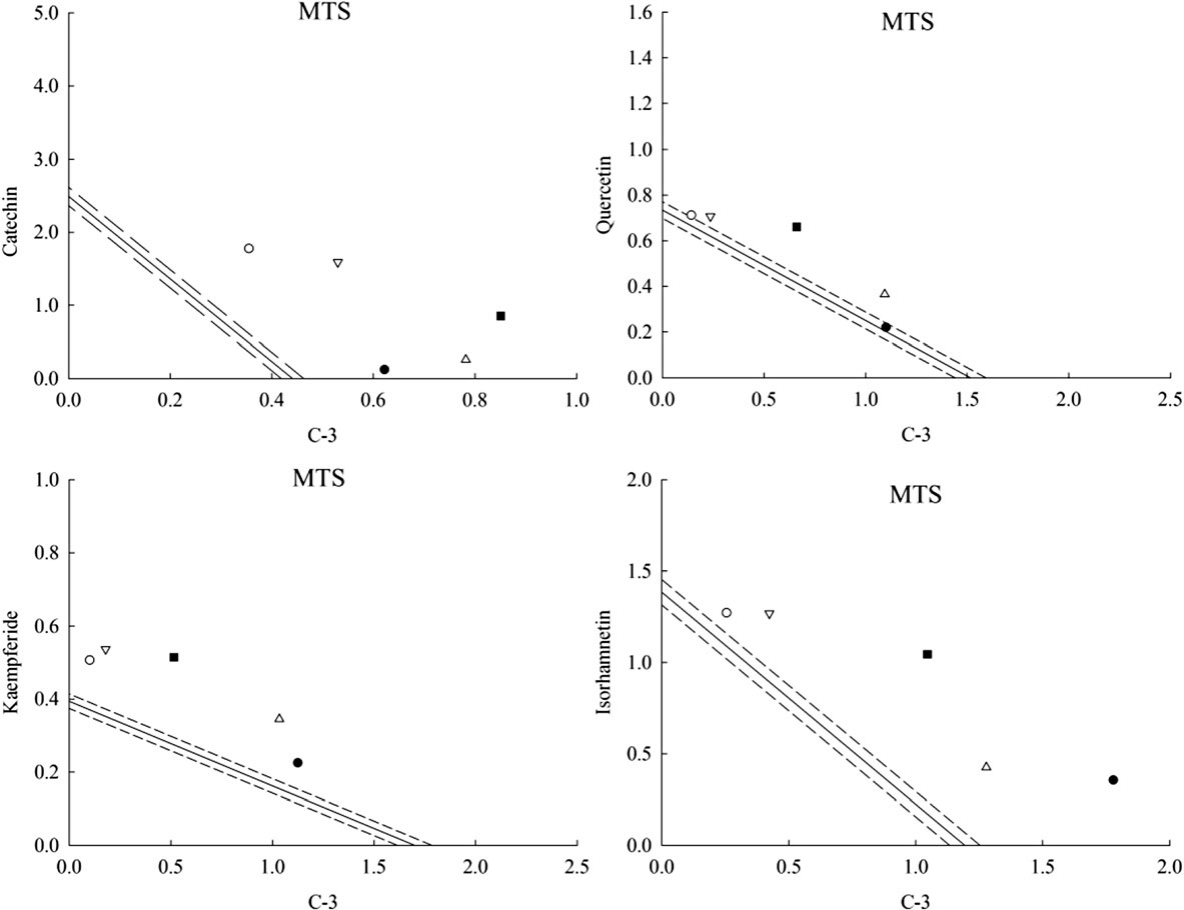
Isobolographic plot of C-3 + catechin, C-3 + quercetin, C-3 + kaempferide and C-3 + isorhamnetin on MTS antioxidant activity

Isobolographic plot of the combinations of C-3 with 4 compounds in EGb was showed in Fig. 3. The observed antioxidant capacity of each mixture was compared with the expected value (Table 1) which was based on the isobologram. In general, no antagonistic effect was found in testing of the combinations and the combination of C-3 + isorhamnetin displayed obvious synergism followed by 1:1 (γ = 1.622) > 5:1 (γ = 1.561) > 3:1 (γ = 1.372) > 1:3 (γ = 1.265) > 1:5 (γ = 1.128).

**Table 1:** Synergistic effects of C-3 + catechin, C-3 + quercetin, C-3 + kaempferide and C-3 + isorhamnetin on MTS antioxidant activity

Combinations	Ratios	Test on growth inhibition		
		O	E	γ
C-3 + Catechin	5:1	0.746 ± 0.019	0.783 ± 0.008	0.954
	3:1	1.043 ± 0.010	0.954 ± 0.009	1.094
	1:1	1.701 ± 0.015*	1.467 ± 0.019	1.159
	1:3	2.118 ± 0.013	1.981 ± 0.012	1.069
	1:5	2.131 ± 0.016	2.152 ± 0.013	0.990
C-3 + Quercetin	5:1	1.320 ± 0.017	1.367 ± 0.017	0.966
	3:1	1.459 ± 0.083	1.323 ± 0.013	1.102
	1:1	1.321 ± 0.010	1.127 ± 0.013	1.172
	1:3	0.942 ± 0.012*	0.931 ± 0.017	1.012
	1:5	0.854 ± 0.017	0.865 ± 0.010	0.987
C-3 + Kaempferide	5:1	1.350 ± 0.006*	1.367 ± 0.008	0.987
	3:1	1.379 ± 0.087	1.375 ± 0.055	1.003
	1:1	1.030 ± 0.018	1.048 ± 0.020	0.983
	1:3	0.716 ± 0.013	0.721 ± 0.015	0.994
	1:5	0.608 ± 0.013	0.612 ± 0.013	0.995
C-3 + Isorhamnetin	5:1	2.134 ± 0.018*	1.367 ± 0.009	1.561
	3:1	1.704 ± 0.020*	1.242 ± 0.021	1.372
	1:1	2.093 ± 0.017*	1.290 ± 0.012	1.622
	1:3	1.693 ± 0.015*	1.338 ± 0.014	1.265
	1:5	1.528 ± 0.013*	1.354 ± 0.016	1.128

Values are the mean of three replicates ± SD (*n* = 3)

The asterisk indicates a significant difference between observed value and expected value (*p* < 0.05). O, observed value; E, expected value; γ, interaction index

Accordingly, these results indicated that there was no obvious synergistic effect among C-3 and other 3 compounds, thus isorhamnetin may played an important role in the synergy of C-3 and EGb combination. Therefore, isorhamnetin were chosen as the specific compounds for further explore the synergistic effects of 6 compounds in C-3 with EGb to find specific combinations with least concentrations that exhibit synergistic effects of C-3 combined with EGb.

### Synergistic effects of 6 compounds in C-3 with isorhamnetin on MTS antioxidant activity

Similarly, to assess how interactions of C-3 and isorhamnetin contribute to the synergistic protective effects, isorhamnetin was added to the other 6 compounds in C-3 on MTS and the protective effect of the combinations was detected.

**Fig. 4:**
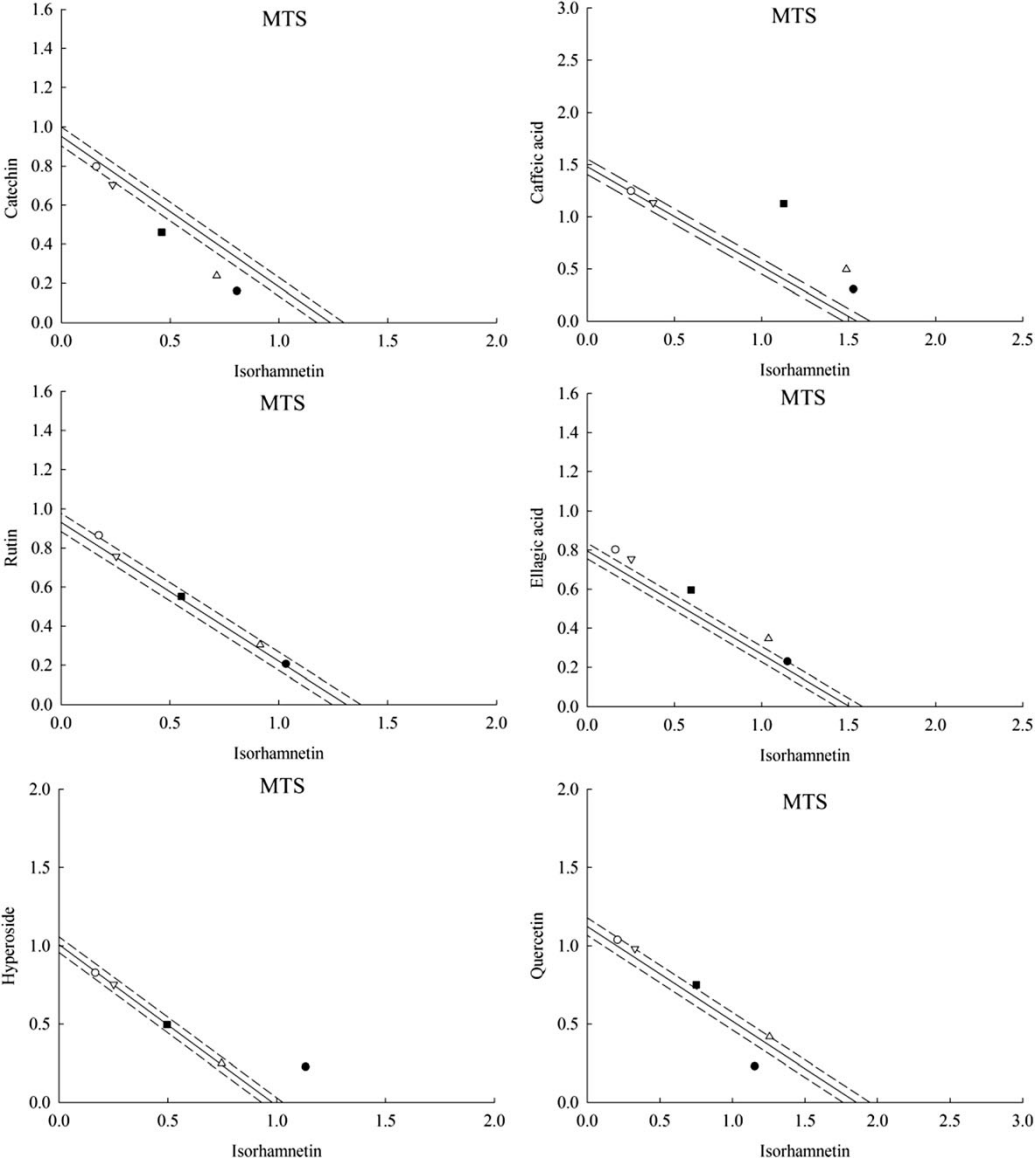
Isobolographic plot of isorhamnetin + catechin, isorhamnetin + caffeic acid, isorhamnetin + rutin, isorhamnetin + ellagic acid, isorhamnetin + hyperoside, isorhamnetin + quercetin on MTS antioxidant activity

The results in Table 2 and Fig. 4 illustrated that majority compounds in C-3 combined with isorhamnetin just showed simple additive effect, there was no significant difference between observed values and expected values (*p* < 0.05). However, the combination of isorhamnetin + caffeic acid showed obvious synergism and the ratio 1:1 exhibited the strongest antioxidant activity on MTS (γ = 1.488) followed by 3:1 (γ = 1.295) > 5:1 (γ = 1.192) > 1:3 (γ = 1.009) > 1:5 (γ = 1.004).

**Table 2:** Synergistic effects of isorhamnetin + catechin, isorhamnetin + caffeic acid, isorhamnetin + rutin, isorhamnetin + ellagic acid, isorhamnetin + hyperoside and isorhamnetin + quercetin on MTS antioxidant activity

Combinations	Ratios	Test on growth inhibition		
		O	E	γ
Isorhamnetin + Catechin	5:1	0.967 ± 0.012*	1.189 ± 0.014	0.813
	3:1	0.952 ± 0.016*	1.165 ± 0.018	0.817
	1:1	0.921 ± 0.013*	1.094 ± 0.012	0.841
	1:3	0.938 ± 0.008*	1.023 ± 0.010	0.917
	1:5	0.957 ± 0.016*	0.999 ± 0.009	0.958
Isorhamnetin + Caffeic acid	5:1	1.834 ± 0.002*	1.538 ± 0.003	1.192
	3:1	1.984 ± 0.018*	1.532 ± 0.018	1.295
	1:1	2.254 ± 0.037*	1.515 ± 0.035	1.488
	1:3	1.511 ± 0.016*	1.497 ± 0.011	1.009
	1:5	1.497 ± 0.015*	1.491 ± 0.020	1.004
Isorhamnetin + Rutin	5:1	1.239 ± 0.009	1.248 ± 0.010	0.993
	3:1	1.218 ± 0.013	1.217 ± 0.016	1.001
	1:1	1.103 ± 0.012	1.122 ± 0.013	0.983
	1:3	1.011 ± 0.011	1.027 ± 0.011	0.984
	1:5	1.039 ± 0.012*	0.996 ± 0.013	1.043
Isorhamnetin + Ellagic acid	5:1	1.379 ± 0.015	1.389 ± 0.010	0.993
	3:1	1.386 ± 0.009	1.330 ± 0.007	1.042
	1:1	1.192 ± 0.009	1.152 ± 0.008	1.035
	1:3	1.007 ± 0.009	0.973 ± 0.013	1.035
	1:5	0.963 ± 0.015	0.914 ± 0.010	1.053
Isorhamnetin + Hyperoside	5:1	1.360 ± 0.015	1.367 ± 0.016	0.995
	3:1	0.996 ± 0.011	0.987 ± 0.009	1.009
	1:1	0.992 ± 0.007	0.994 ± 0.009	0.999
	1:3	1.004 ± 0.008	1.000 ± 0.010	1.004
	1:5	0.993 ± 0.010	1.002 ± 0.010	0.991
Isorhamnetin + Quercetin	5:1	1.384 ± 0.014	1.367 ± 0.014	1.012
	3:1	1.674 ± 0.008	1.673 ± 0.007	1.001
	1:1	1.501 ± 0.009*	1.490 ± 0.015	1.008
	1:3	1.310 ± 0.015	1.307 ± 0.010	1.002
	1:5	1.247 ± 0.086	1.246 ± 0.001	1.001

Values are the mean of three replicates ± SD (*n* = 3)

The asterisk indicates a significant difference between observed value and expected value (*p* < 0.05). O, observed value; E, expected value; γ, interaction index

In conclusion, the observation suggested that the synergistic protective effect response in C-3 + EGb may be due to the interacting of isorhamnetin and caffeic acid. Furthermore, the combinations of C-3 + EGb and isorhamnetin + caffeic acid were selected to reveal the related bioactive mechanism behind this synergism. Moerover, the ratio of 1:1 (isorhamnetin: caffeic acid) may be chosen as the best ratio that exhibited the synergistic effects in the combination of C-3 and EGb.

### Influence of C-3 + EGb and isorhamnetin + caffeic acid on H_2_O_2_ antioxidant production rate

Previous research showed that the antioxidant capacity of the combinations and their autoxidation of H_2_O_2_ producing ability are proportional to the relationship [28]. The results in Table 3 showed that the combination of C-3 + EGb generally displayed an additive effect while 5:1 displayed synergism (γ = 1.192). Furthermore, isorhamnetin + caffeic acid showed a trend of synergism in which 1:1 displayed the strongest synergism (γ = 1.536). This suggested that the presence of isorhamnetin and caffeic acid played a great role in the synergy observed.

**Table 3:** H_2_O_2_ production rate of C-3 + EGb and isorhamnetin + caffeic acid

H_2_O_2_ production Rate (μM/min)	Ratio	O	E	γ
C-3 + EGb	5:1	0.339 ± 0.013*	0.285 ± 0.014	1.192
	3:1	0.300 ± 0.019	0.278 ± 0.013	1.080
	1:1	0.274 ± 0.014	0.257 ± 0.012	1.068
	1:3	0.252 ± 0.013	0.236 ± 0.014	1.067
	1:5	0.238 ± 0.013	0.229 ± 0.014	1.041
Isorhamnetin + Caffeic acid	5:1	0.481 ± 0.001*	0.319 ± 0.003	1.509
	3:1	0.490 ± 0.001*	0.321 ± 0.002	1.524
	1:1	0.503 ± 0.001*	0.327 ± 0.001	1.536
	1:3	0.444 ± 0.003*	0.334 ± 0.001	1.331
	1:5	0.399 ± 0.002*	0.336 ± 0.001	1.189

Values are the mean of three replicates ± SD (*n* = 3)

The asterisk indicates a significant difference between observed value and expected value (*p* < 0.05). O, observed value; E, expected value; γ, interaction index

Among the results detected, the combination of C-3 + EGb and isorhamnetin + caffeic acid showed synergistic on H_2_O_2_ antioxidant production rate, which was in accordance with the results above on MTS. It was suggested that the synergism response in C-3 + EGb may be due to the interacting of isorhamnetin and caffeic acid, thus the combination of C-3 + EGb and isorhamnetin + caffeic acid were picked out to in-depth explore the related bioactive mechanism behind synergy through the determination of enzymatic antioxidant activites in *E. coli*.

### Influence of C-3 + EGb and isorhamnetin + caffeic acid on CAT, POD, SOD and GSH enzyme activities

To ascertain the root cause why the combinations above promoted the protective effect on MTS, the activities of four main antioxidant enzymes (CAT, POD, SOD and GSH-PX) in *E. coli* were determined. Results showed that both the combinations of C-3 + EGb and isorhamnetin + caffeic acid promoted CAT and SOD enzyme activities and the proportion of 1:1 exhibited the strongest effect with value of 13.175 ± 0.071 U/mg prot, 105.125 ± 0.073 U/mg prot, 10.699 ± 0.101 U/mg prot and 125.970 ± 1.681 U/mg prot (Table 4). However, these two combinations did not affect much on POD and GSH enzyme activities. All these experimental results above highlighted that synergy response in the combination of C-3 + EGb had a correlation with isorhamnetin + caffeic acid.

**Table 4:** Influence of C-3 + EGb and isorhamnetin + caffeic acid on CAT, POD, SOD and GSH enzyme activities

Combinations	Ratios	CAT activity U/mg prot			POD activity U/mg prot			SOD activity U/mg prot			GSH activity U/mg prot		
		O	E	γ	O	E	γ	O	E	γ	O	E	γ
C-3 + EGb	5:1	10.578 ± 0.067	10.613 ± 0.085	0.997	84.837 ± 1.901	83.467 ± 0.900	1.016	90.775 ± 1.038	88.600 ± 1.257	1.025	114.376 ± 1.697	113.692 ± 1.529	1.006
	3:1	10.465 ± 0.058*	10.936 ± 0.073	0.957	80.115 ± 0.847	79.996 ± 0.755	1.001	98.150 ± 0.051	85.525 ± 1.551	1.148	110.222 ± 1.880	109.027 ± 1.956	1.011
	1:1	13.175 ± 0.071*	11.908 ± 0.066	1.106	78.015 ± 0.798	69.581 ± 0.724	1.121	105.125 ± 0.073*	76.325 ± 1.356	1.377	102.312 ± 1.587	95.031 ± 1.365	1.077
	1:3	12.883 ± 0.120	12.880 ± 0.090	1.000	59.346 ± 0.736	59.166 ± 0.686	1.003	66.500 ± 1.112	67.125 ± 1.193	0.991	82.964 ± 1.373	81.036 ± 1.703	1.024
	1:5	13.315 ± 0.083	13.204 ± 0.068	1.008	57.514 ± 0.681	55.695 ± 0.949	1.033	67.800 ± 0.178	64.050 ± 1.138	1.058	78.2775 ± 1.541	76.371 ± 1.143	1.025
Isorhamneti + Caffeic acid	5:1	9.297 ± 0.054	8.878 ± 0.079	1.047	77.455 ± 0.618	72.404 ± 0.610	1.070	107.130 ± 1.690	109.134 ± 1.772	0.982	102.798 ± 1.105	99.666 ± 0.776	1.032
	3:1	8.445 ± 0.078	8.520 ± 0.069	0.991	85.398 ± 0.759	83.218 ± 0.675	1.026	107.418 ± 1.438	103.410 ± 1.858	1.039	96.300 ± 1.230	95.760 ± 0.549	1.006
	1:1	10.699 ± 0.101*	7.445 ± 0.066	1.437	119.896 ± 0.538	115.659 ± 0.871	1.037	125.970 ± 1.681*	86.244 ± 1.112	1.461	82.170 ± 0.776	84.078 ± 1.074	0.977
	1:3	6.729 ± 0.067	6.371 ± 0.082	1.056	164.424 ± 0.620	148.100 ± 0.670	1.110	71.928 ± 1.925	69.078 ± 1.368	1.041	74.412 ± 0.680	72.396 ± 1.187	1.028
	1:5	6.729 ± 0.064	6.013 ± 0.063	1.119	160.563 ± 0.386	158.913 ± 0.322	1.010	62.376 ± 1.673	63.360 ± 1.453	0.985	69.966 ± 0.845	68.490 ± 1.192	1.021

Values are the mean of three replicates ± SD (*n* = 3)

The asterisk indicates a significant difference between observed value and expected value (*p* < 0.05). O, observed value; E, expected value; γ, interaction index

**Fig. 5:**
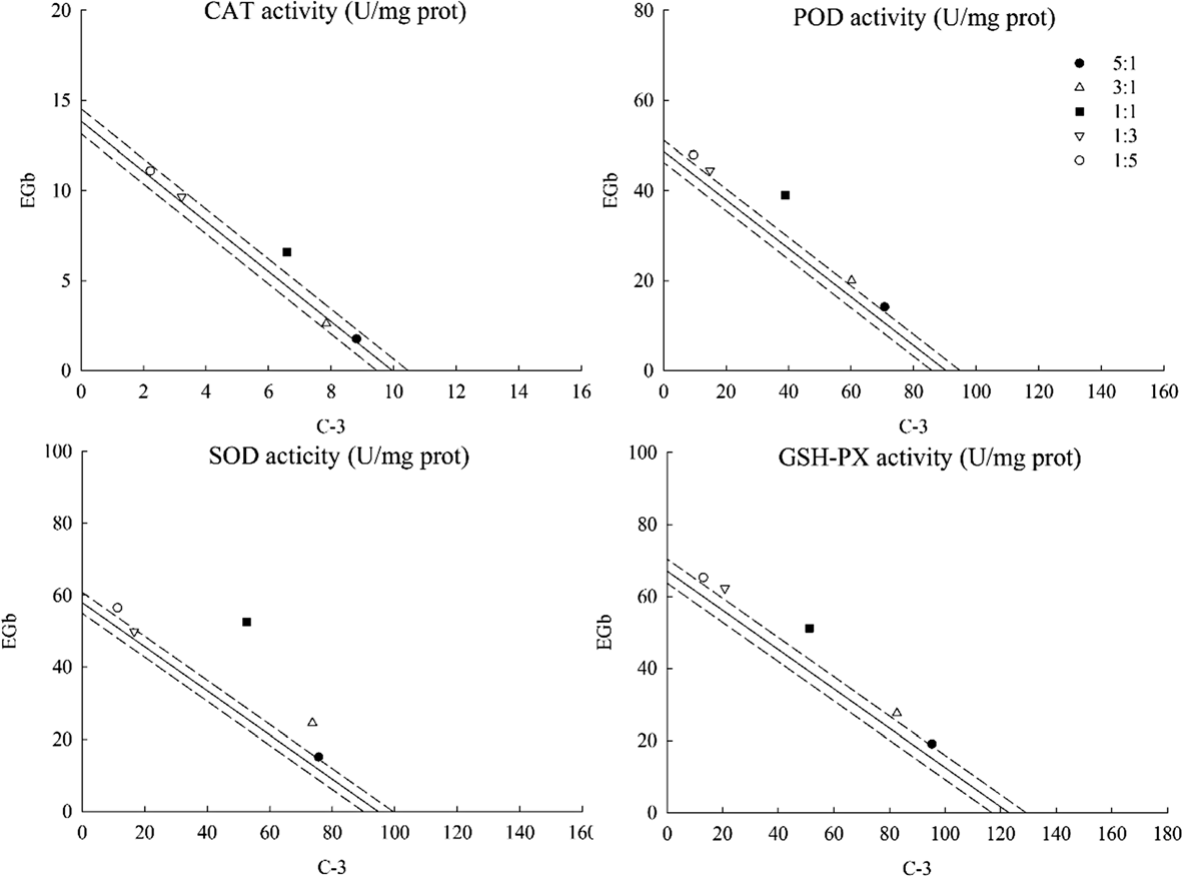
Isobolographic plot of C-3 + EGb on CAT, POD, SOD and GSH-PX enzyme activities

**Fig. 6:**
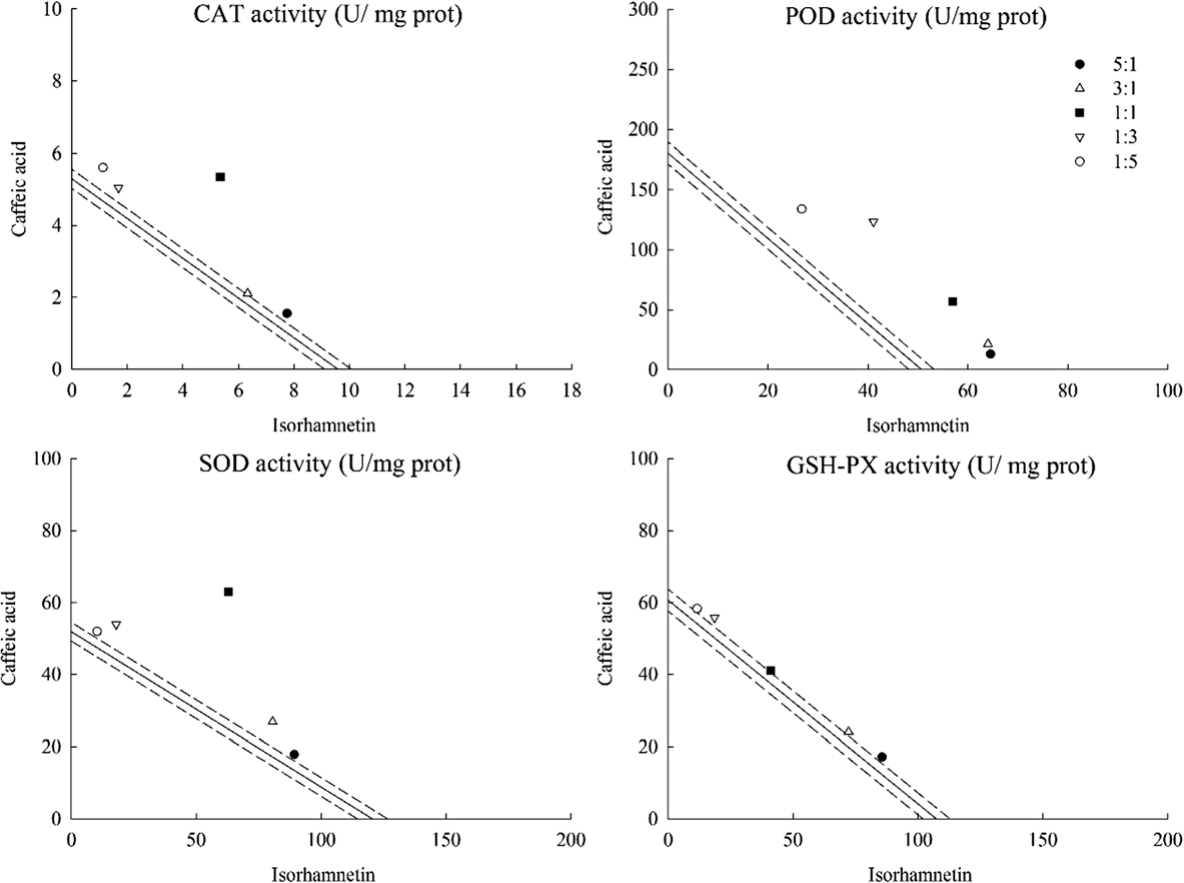
Isobolographic plot of isorhamnetin + caffeic acid on CAT, POD, SOD and GSH-PX enzyme activities

In addition, the combinations of C-3 + EGb and isorhamnetin + caffeic acid were evaluated for their antioxidant capacities as isobolographic plots showed in Figs. 5 and 6. Accordingly, the combination of isorhamnetin + caffeic acid showed obvious synergy in the CAT and SOD enzyme activity at the ratio 1:1, suggesting that isorhamnetin and caffeic acid may be the main compounds that affected the enzyme activities in *E. coli*.

Similar to the results in previous chapter, other ratios of the two combinations showed simple additive effects, which could be speculated that the related bioactive mechanism of combined compounds closely linked to the effects of antioxidant enzyme activities.

### Influence of C-3 + EGb and isorhamnetin + caffeic acid on CAT, SOD_a_, SOD_b_ and SOD_c_ gene expression by real-time PCR

To reveal the reason why the combinations of C-3 + EGb and isorhamnetin + caffeic acid promoted CAT and SOD enzyme activities, influence on CAT, SOD_a_, SOD_b_ and SOD_c_ gene expression were measured by real-time PCR. Among the results, C-3 + EGb and isorhamnetin + caffeic acid promoted CAT and SOD_a_ gene expression and the ratio 1:1 of two combinations exhibited the strongest promotion with value of 1.279 ± 0.140, 1.569 ± 0.045, 1.395 ± 0.004 and 1.685 ± 0.019 (γ = 1.122, 1.275, 1.075 and 1.211) respectively (Table 5). However, the combinations of C-3 + EGb and isorhamnetin + caffeic acid did not promote SOD_b_ and SOD_c_ gene expression so significant.

**Table 5:** Influence of C-3 + EGb and isorhamnetin + caffeic acid on CAT, SOD_a_, SOD_b_ and SOD_c_ gene expression

Combinations	Ratios	CAT			SOD_a_			SOD_b_			SOD_c_		
		O	E	γ	O	E	γ	O	E	γ	O	E	γ
C-3 + EGb	5:1	1.070 ± 0.029	1.102 ± 0.019	0.971	1.158 ± 0.026	1.031 ± 0.026	1.123	1.301 ± 0.012	1.370 ± 0.013	0.949	1.409 ± 0.063	1.394 ± 0.021	1.011
	3:1	1.100 ± 0.071*	1.112 ± 0.014	0.990	1.027 ± 0.016	1.081 ± 0.034	0.950	1.322 ± 0.044	1.384 ± 0.019	0.955	1.423 ± 0.030	1.409 ± 0.013	1.010
	1:1	1.279 ± 0.140*	1.141 ± 0.023	1.122	1.569 ± 0.045	1.231 ± 0.059	1.275	1.528 ± 0.020	1.424 ± 0.038	1.073	1.522 ± 0.070	1.454 ± 0.054	1.047
	1:3	1.022 ± 0.006	1.170 ± 0.025	0.874	1.207 ± 0.023	1.380 ± 0.083	0.874	1.428 ± 0.081	1.464 ± 0.056	0.976	1.457 ± 0.076	1.499 ± 0.083	0.972
	1:5	1.028 ± 0.011	1.180 ± 0.028	0.871	1.334 ± 0.022	1.430 ± 0.091	0.933	1.443 ± 0.010	1.478 ± 0.063	0.977	1.501 ± 0.071	1.514 ± 0.025	0.991
Isorhamnetin + Caffeic acid	5:1	1.283 ± 0.065	1.289 ± 0.025	0.995	1.220 ± 0.016	1.289 ± 0.013	0.946	1.206 ± 0.027	1.183 ± 0.025	1.020	1.342 ± 0.047	1.347 ± 0.046	0.996
	3:1	1.264 ± 0.018	1.292 ± 0.049	0.979	1.300 ± 0.008	1.315 ± 0.011	0.989	1.135 ± 0.021	1.171 ± 0.026	0.969	1.348 ± 0.033	1.338 ± 0.004	1.007
	1:1	1.395 ± 0.004	1.298 ± 0.012	1.075	1.685 ± 0.019*	1.391 ± 0.049	1.211	1.289 ± 0.040	1.135 ± 0.028	1.135	1.477 ± 0.020*	1.310 ± 0.018	1.127
	1:3	1.352 ± 0.047	1.305 ± 0.019	1.036	1.467 ± 0.009	1.467 ± 0.011	1.000	1.080 ± 0.017	1.100 ± 0.030	0.982	1.227 ± 0.018	1.282 ± 0.027	0.957
	1:5	1.321 ± 0.002	1.307 ± 0.021	1.011	1.512 ± 0.025	1.492 ± 0.031	1.013	1.109 ± 0.006	1.088 ± 0.031	1.019	1.233 ± 0.021	1.273 ± 0.089	0.968

Values are the mean of three replicates ± SD (*n* = 3)

The asterisk indicates a significant difference between observed value and expected value (*p* < 0.05). O, observed value; E, expected value; γ, interaction index

**Fig. 7:**
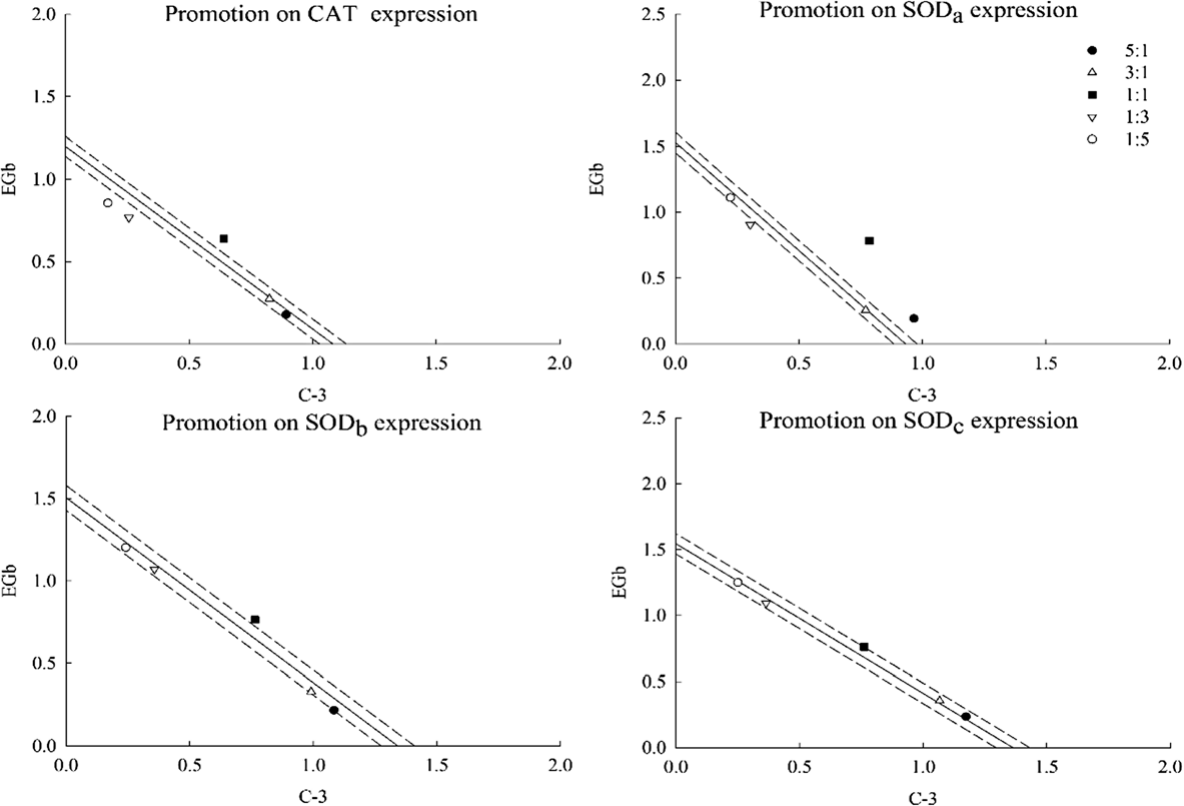
Influence of C-3 + EGb on CAT, SOD_a_, SOD_b_ and SOD_c_ gene expression

**Fig. 8:**
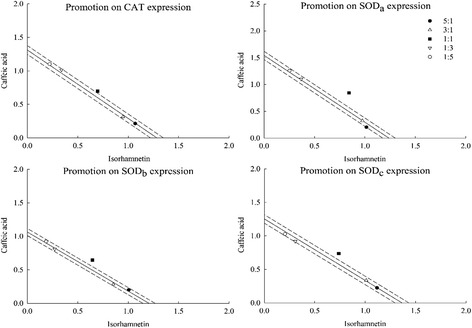
Influence of isorhamnetin + caffeic acid on CAT, SOD_a_, SOD_b_ and SOD_c_ gene expression

Similarly, isobolographic plot of C-3 + EGb and isorhamnetin + caffeic acid on CAT, SOD_a_, SOD_b_ and SOD_c_ gene expression both showed significant synergistic promoting effect on CAT and SOD_a_ gene expression and the synergy was most significant at the ratio 1:1 (Figs. 7 and 8). The results also showed that the combination of C-3 + EGb just displayed an additive effect in SOD_b_ and SOD_c_ gene expression as well as the combination of isorhamnetin + caffeic acid.

In conclusion, the changes in gene expression may be closely linked to those in enzyme activity. The results showed the combination of isorhamnetin + caffeic acid promoted CAT and SOD_a_ gene expression and had no obvious promoting effect on SOD_b_ and SOD_c_ gene expression. This further explain that the synergistic effects of C-3 combined with EGb may have relations with its components of isorhamnetin and caffeic acid. Interactions between isorhamnetin and caffeic acid may played an important role in the related bioactive mechanism behind synergism. However, the problem still needs further research.

## Conclusions

In conclusion, the combination of C-3 + EGb in various ratios exhibited significant synergistic effect on MTS and the ratio 5:1(C-3: EGb) showed the strongest synergy. Similarly, the combination of isorhamnetin + caffeic acid also displayed synergistic effect on MTS and the ratio 1:1 exhibited the strongest synergy. These results were in accordance with that determined in H_2_O_2_ antioxidant production rate. Furthermore, both combinations of C-3 + EGb and isorhamnetin + caffeic acid promoted CAT and SOD enzyme activities and the ratio 1:1 exhibited the strongest synergy. What’s more, both combinations above promoted gene expression of CAT and SOD enzyme and the ratio 1:1 exhibited the strongest synergy. Therefore, the related bioactive mechanism behind the synergism of C-3 combined with EGb may be attributed to the combination of isorhamnetin and caffeic acid which promoted gene expression of CAT and SOD enzyme as well as further promoted CAT and SOD enzyme activities thus showed synergistic effect.

*P. fruticosa* extracts have long been used for its superior therapeutic in traditional Tibetan medicine in China. This study provided relevant theoretical basis for maximizing biological antioxidant capacity and physiological bioavailability of *P. fruticosa* using EGb in lower doses to avoid the side effects. Moreover, isorhamnetin + caffeic acid could be used as the specific combination that exhibit synergistic effect of *P. fruticosa* combined with EGb. These results could also be useful to elaborate the related bioactive mechanism behind the synergy of *P. fruticosa* combined with EGb for theoretical guidance to develop new medicines and natural products. Nevertheless, more researches need to be conducted to explain fundamental reason.

## Abbreviations

ANOVA: Analysis of variance
C-3: *P. fruticosa* leaf extracts (the acetone extraction phase)
CAT: Catalase enzyme
EGb: *Ginkgo biloba* extracts
GSH-PX: Glutathione peroxidase enzyme
MTS: Microbial test system
POD: Peroxidase enzyme
SOD: Superoxide dismutase enzyme
